# Sarcopenia-related traits and erectile dysfunction: a bi-directional Mendelian randomization study

**DOI:** 10.1093/sexmed/qfag010

**Published:** 2026-03-27

**Authors:** Xu Jianxin, Wu Wensong, Fang Dengpan, Wang Yiwei, Chen Fangmin

**Affiliations:** Urology, The Sixth Hospital of Wuhan, Affiliated Hospital of Jianghan University, Wuhan 430000, China; Urology, The Sixth Hospital of Wuhan, Affiliated Hospital of Jianghan University, Wuhan 430000, China; Urology, The Sixth Hospital of Wuhan, Affiliated Hospital of Jianghan University, Wuhan 430000, China; Urology, The Sixth Hospital of Wuhan, Affiliated Hospital of Jianghan University, Wuhan 430000, China; Urology, The Sixth Hospital of Wuhan, Affiliated Hospital of Jianghan University, Wuhan 430000, China

**Keywords:** Sarcopenia, erectile dysfunction, Mendelian randomization

## Abstract

**Background:**

Erectile dysfunction (ED) and sarcopenia share many risk factors, but the causal direction of their association remains unclear.

**Aim:**

To investigate the potential causal relationship between sarcopenia-related traits and ED using genetic data.

**Methods:**

We conducted a bi-directional 2-sample Mendelian randomization (MR) analysis to evaluate the potential causal relationship between sarcopenia-related traits (hand grip strength, appendicular lean mass, and walking pace) and ED. We selected the inverse variance weighted (IVW) method as the main method to assess the causal effect, and we use Cochrane’s Q tests derived from the IVW and MR-Egger method to evaluate the heterogeneity. To investigate horizontal pleiotropy, the study employed MR-Egger and MR-PRESSO methods. Leave-one-out analysis was conducted to assess the influence of individual genetic loci on the outcomes.

**Outcomes:**

Specific sarcopenia-related functional traits might increase the risk of ED, while reverse causality was not observed.

**Results:**

When considering sarcopenia-related characteristics as exposure factors, we found a positive causal relationship between appendicular lean mass and ED (OR = 1.097, CI = 1.009-1.194, *P* = .029) and a negative causal relationship between walking pace and ED (OR = 0.325, CI = 0.172-0.613, *P* = .00052). Hand grip strength showed no causal relationship with ED. Reverse MR results indicated that ED as an exposure factor did not have a causal relationship with sarcopenia-related characteristics. The MR-Egger intercept indicated no evidence of horizontal pleiotropy (*P* > .05). Cochran’s Q test showed no significant heterogeneity (*P* > .05). Leave-one-out analysis revealed that excluding any single SNP did not significantly change the overall error lines, confirming the reliability of our conclusions.

**Clinical Implications:**

Interventions aimed at improving overall physical function and fitness, particularly addressing a slow walking pace, may help reduce the risk of ED.

**Strengths and Limitations:**

Key strengths include the MR design minimizing confounding and reverse causation, and comprehensive sensitivity analyses. The main limitation is that our study only contains samples of European descent.

**Conclusion:**

Specific sarcopenia-related functional traits, particularly a slower walking pace, may play a causal role in ED. These findings highlight the importance of overall physical function and fitness in ED risk, although further studies are needed to clarify the specific role of muscle mass.

## Introduction

Erectile dysfunction (ED) is defined as the inability of the patient to achieve or maintain sufficient penile erection to allow for normal sexual intercourse. Many patients feel inferior and ashamed due to their condition, which can lead to negative emotions and increase the risk of psychological disorders over time.^1^ Men over the age of 40 are at a higher risk for ED.^2^ Globally, approximately 150 million men are affected by ED.^3^ Major risk factors for ED include smoking, alcohol consumption, unhealthy diet, cardiovascular diseases, and lack of exercise.^4^ With the prevalence of these risk factors, the incidence of ED remains consistently high.

Sarcopenia is a complex, multifactorial disease characterized by the reduction of skeletal muscle mass, muscle strength, and physical function.^5^ This condition is prevalent among the elderly population and is influenced by lifestyle, disease, and genetic factors.^6^ The concept of sarcopenia was first introduced in 1989 by Irwin Rosenberg from Tufts University in the United States, to describe the age-related decline in muscle mass.^7^ In 2019, the European Working Group on Sarcopenia in Older People (EWGSOP) further emphasized that sarcopenia encompasses not only the reduction in muscle mass but also the decline in muscle strength and physical function.^8^

Sarcopenia and ED are both associated with aging, inflammation, and endocrine dysregulation, such as a decline in androgen levels. Sarcopenia may be linked to low androgen levels, as reduced androgens can lead to decreased anabolic processes in skeletal muscle.^9^ Consequently, androgen supplementation has been proposed as a treatment for sarcopenia.^10^ In a population-based study of elderly men in a Korean community, the prevalence of severe ED and sarcopenia was found to be 52.4% and 31.6%, respectively.^11^ Cross-sectional analysis revealed that men with sarcopenia were more likely to suffer from severe ED compared to those without sarcopenia.^11^ Although several observational studies suggest a potential link between ED and sarcopenia, there is currently a lack of systematic research evaluating the causal relationship between sarcopenia and ED.

In traditional observational epidemiological studies, the relationship between exposure and outcome is often influenced by confounding factors and reverse causality, and these studies are typically time-consuming, labor-intensive, and require long observation periods. Mendelian randomization (MR) is a method akin to a natural randomized controlled trial, which uses genetic variants, specifically single nucleotide polymorphisms (SNPs), as instrumental variables to infer causal relationships between exposure and outcome.^12^ Since genetic variants are randomly assigned at conception, this approach can mitigate the influence of confounding factors such as behavior and environment.^13^ Therefore, this study employs MR analysis to answer the following question: Is there a causal relationship between sarcopenia-related traits and the risk of erectile dysfunction?

## Methods

### Study design

**Figure 1:**
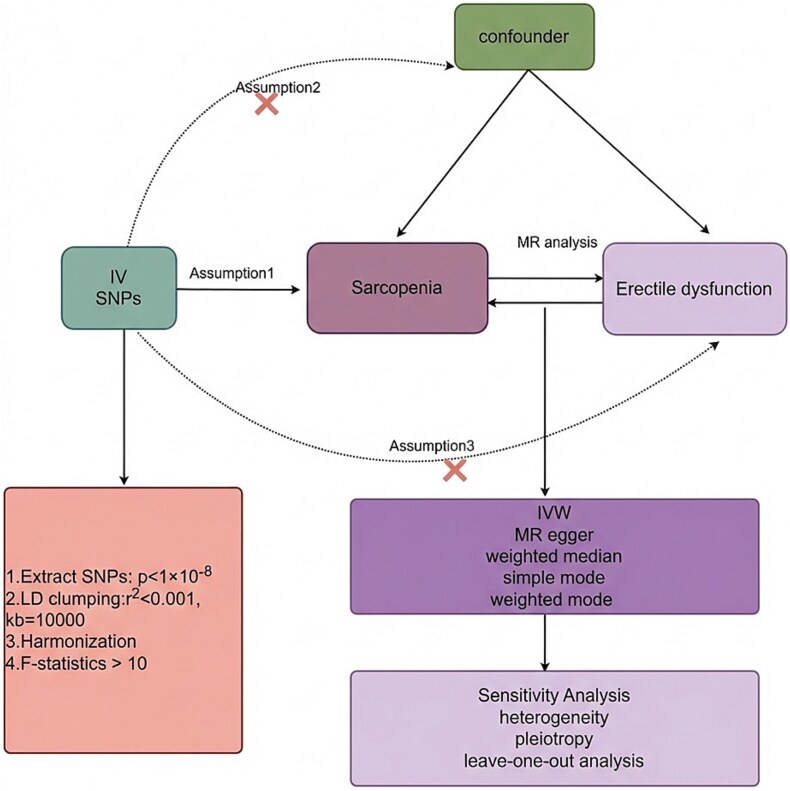
Study flowchart.

This study employed a bi-directional MR approach to investigate the causal relationship between sarcopenia and ED. The MR analysis was based on three fundamental assumptions: (1) there must be a strong association between SNPs and the exposure, (2) SNPs should not be associated with potential confounders (*P* > .05), and (3) the effects of SNPs on the outcome should be mediated solely through the exposure. Figure 1 provides a detailed overview of the study methodology. The detailed methodology of this study is illustrated in Figure 1.

### Data acquisition

Sarcopenia-related traits include appendicular lean mass (ALM), hand grip strength, and walking pace. The summary data for ALM, derived from a European cohort, were measured using bioelectrical impedance analysis and adjusted for parameters such as age and gender, including 450 243 European individuals. The summary data for ALM were measured using bioelectrical impedance analysis and adjusted for para and is the most used proxy for muscle mass in sarcopenia research.^14^ Hand grip strength, widely utilized as an indicator of muscle health, provides a simple and non-invasive method to measure general muscle strength.^15^ The hand grip strength data were obtained from 48 596 European individuals. According to the practical clinical definition and consensus diagnosis criteria for sarcopenia established by the EWGSOP, sarcopenia is diagnosed when reduced muscle mass is accompanied by decreased muscle strength and/or impaired physical function (walking speed ≤0.8 m/s).^16^ The walking pace Genome-wide association study (GWAS) data were collected from 459 915 individuals of European descent. Additionally, GWAS summary data for ED were obtained from the UK Biobank, and includes 223 805 European samples collected in 2018, comprising 6175 ED cases and 217 630 control samples.

### Selection of instrumental variables

When selecting IVs in MR analysis, several key principles are followed. First, the significance threshold was set at *P* < 5 × 10^−8^ to ensure that the selected SNPs were significantly associated with micronutrients. Second, the LD clustering algorithm in R software is used to reduce the correlation and linkage disequilibrium between instrumental variables. This algorithm filters out independent instrumental variables by setting a threshold of *r*^2^ < 0.001 within a 10 000 kb distance. Third, to ensure the consistency and reliability of MR analyses, SNPs data were harmonized, including aligning the allelic orientation of SNPs, excluding palindromic SNPs, and removing SNPs that did not match the exposure and outcome variables. Finally, the F-statistic is calculated to assess its effectiveness as IVs. Generally, IVs with an F-statistic value greater than 10 are considered suitable for MR analysis. The application of these standards ensures that the results of MR studies are both reliable and valid.

### MR analysis

We selected the inverse variance weighted (IVW) method as the main method to assess the causal effect between exposure and outcome, which was the most reliable indicator if there was no evidence of horizontal pleiotropy in the selected IVs.^17^ MR Egger, weighted median, simple model, and weighted model methods were used as supplementary methods.

To enhance the robustness of causal inference, we assessed heterogeneity using the Cochrane’s Q test, with *P* < .05 indicating the presence of heterogeneity. To avoid horizontal pleiotropy, this study used the MR Egger method to detect pleiotropy by testing the residual intercept of the MR model. In addition, leave-one-out analysis was used to assess the impact of a single gene locus on the outcome to ensure the robustness and reliability of the model. All analyses were performed using R software (version 4.5.0, R Foundation for Statistical Computing, Vienna, Austria).

## Results

**Figure 2:**
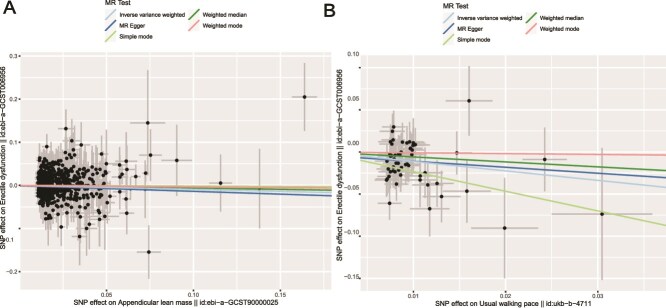
Scatter plots of MR analyses. (A) Scatter plot of genetic correlation between the ALM and ED; (B) scatter plot of genetic correlation between the walking pace and ED.

Following the above methods, we identified 57 SNPs related to walking pace, 629 SNPs related to ALM, 17 SNPs related to hand grip strength, and 176 SNPs related to ED. The F-statistics for all selected IVs were greater than 10, indicating that no weak instruments were included in the study. When considering sarcopenia-related characteristics as exposure factors, we found a positive causal relationship between ALM and ED (OR = 1.097, CI = 1.009-1.194, *P* = .029) (Figure 2A) (Table 1) and a negative causal relationship between walking pace and ED (OR = 0.325, CI = 0.172-0.613, *P* = .00052) (Figure 2B) (Table 1). Hand grip strength showed no causal relationship with ED. Reverse MR results indicated that ED as an exposure factor did not have a causal relationship with sarcopenia-related characteristics (Table 2). To avoid bias, we conducted several sensitivity analyses to test the reliability of the MR results and detect potential horizontal pleiotropy. The MR-Egger intercept indicated no evidence of horizontal pleiotropy (*P* > .05). Cochran’s Q test showed no significant heterogeneity (*P* > .05) (Table 3). Leave-one-out analysis revealed that excluding any single SNP did not significantly change the overall error lines (all error lines remained to the right of 0), confirming the reliability of our conclusions (Figure S1). These findings suggested that sarcopenia might increase the risk of ED, while having ED does not increase the risk of sarcopenia.

**Table 1:** Mendelian randomization estimates for sarcopenia on ED.

Outcome	Exposure	Method	OR	95% CI	*P*-value
Erectile dysfunction	Appendicular lean mass	MR Egger	1.138	0.937-1.381	.191
		Weighted median	1.064	0.926-1.221	.377
		IVW	1.097	1.009-1.194	**.029**
		Simple mode	1.084	0.642-1.508	.943
		Weighted mode	1.035	0.783-1.367	.807
Erectile dysfunction	Hand grip strength	MR Egger	0.951	0.486-1.858	.886
		Weighted median	0.961	0.722-1.280	.789
		IVW	0.894	0.724-1.103	.298
		Simple mode	1.046	0.615-1.779	.867
		Weighted mode	1.036	0.630-1.705	.888
Erectile dysfunction	Walking pace	MR Egger	0.491	0.036-6.552	.593
		Weighted median	0.554	0.221-1.389	.208
		IVW	0.325	0.172-0.613	**.00052**
		Simple mode	0.097	0.011-0.810	**.035**
		Weighted mode	0.903	0.169-7.199	.918

Bold values indicate statistical significance.

**Table 2:** Mendelian randomization estimates for ED on sarcopenia.

Outcome	Exposure	Method	OR	95% CI	*P*-value
Appendicular lean mass	Erectile dysfunction	MR Egger	0.968	0.927-1.010	.145
		Weighted median	0.989	0.967-1.012	.375
		IVW	0.987	1.009-1.194	.406
		Simple mode	0.891	0.959-1.016	.990
		Weighted mode	0.891	0.733-1.327	.990
Hand grip strength	Erectile dysfunction	MR Egger	0.961	0.871-1.059	.429
		Weighted median	1.050	0.968-1.140	.236
		IVW	0.977	0.910-1.037	.396
		Simple mode	0.876	1.213-1.779	.990
		Weighted mode	0.876	0.430-1.305	.991
Walking pace	Erectile dysfunction	MR Egger	0.993293	0.976-1.010	.435
		Weighted median	0.998532	0.984-1.012	.834
		IVW	0.98952	0.978-1.000	.067
		Simple mode	0.956726	0.042-21.308	.977
		Weighted mode	1.018334	0.059-17.468	.990

**Table 3:** Heterogeneity and Horizontal pleiotropy analysis.

Exposure	Outcome	Heterogeneity	Horizontal pleiotropy	MR Egger intercept *P*
		Q_Pvalue (IVW)	Q_Pvalue (MR Egger)	
Erectile dysfunction	Appendicular lean mass	.503	.493	.685
Erectile dysfunction	Hand grip strength	.704	.636	.851
Erectile dysfunction	Walking pace	.703	.673	.748
Appendicular lean mass	Erectile dysfunction	.745	.569	.223
Hand grip strength	Erectile dysfunction	.185	.698	.761
Walking pace	Erectile dysfunction	.362	.146	.551

## Discussion

Many factors contributing to ED and sarcopenia are similar, although they have different underlying mechanisms. Age is a major risk factor for the development of both ED and sarcopenia, with the incidence of these conditions increasing with age.^18^^,^^19^ In healthy populations under the age of 70, the prevalence of sarcopenia is approximately 20%; however, this rate exceeds 50% in individuals over 80 years old.^20^ Among men aged 60-69, the prevalence of ED rises to 20%-40%, while in men over 70, the prevalence can reach 50%-100%.^21^ Although recent studies suggest a potential association between ED and sarcopenia, research exploring this relationship remains limited. In this study, we utilized a bi-directional MR analysis with aggregated data, confirming a causal relationship between sarcopenia-related traits and ED. The results indicate a causal relationship between ALM and walking pace with ED, whereas grip strength does not exhibit a causal relationship with ED.

Our results demonstrated a slight positive causal association between higher ALM and increased ED risk. Although this finding appears counterintuitive to the biological framework where sarcopenia increases ED risk, it can be explained by the distinction between static muscle quantity and dynamic functional quality. ALM is a purely static anatomical measure of muscle mass. In genetic models, instrumental variables predicting higher ALM are frequently associated with a larger overall body size and higher body mass index (BMI). Given that elevated BMI and adiposity are well-established independent risk factors for endothelial dysfunction and vascular ED, this phenotypic confounding may drive the slight increase in ED risk observed. Furthermore, the small effect size underscores that static muscle mass alone is not a definitive predictor of ED compared to functional metrics. Sarcopenia is characterized by age-related declines in muscle mass, strength, and function. A lack of physical activity is considered a primary risk factor for this condition, with muscle fiber and strength deterioration beginning as early as age 50 in sedentary individuals.^6^ Reductions in hormone levels and insulin-like growth factor can lead to muscle mass and strength loss.^22^ Additionally, a combination of pro-inflammatory cytokines can result in sarcopenia.^6^^,^^23^ Previous research has shown that approximately one-third of physically inactive men suffer from ED, whereas those who engage in at least 32.6 metabolic equivalents (METs) of exercise per week have a significantly lower risk of ED compared to men who engage in less than 2.7 METs per week, a risk reduction comparable to the reduction in obesity-related risks.^24^ In one study, 110 obese men with ED were divided into intervention and control groups. The intervention group aimed to reduce body weight by 10% through walking four hours per week. After two years, their International Index of Erectile Function Scores significantly improved, whereas the control group showed no meaningful change. Additionally, the study demonstrated an independent association between physical inactivity and ED.^25^ In a subsequent larger study, patients who exercised more than 4 h per week were 1.9 times more likely to reverse ED than their sedentary counterparts. Furthermore, high-intensity exercise 3 times a week for 60 min significantly improved ED, with these improvements correlating with enhanced physical fitness.^26^ Previous research indicated that leg press strength, leg power, and increases in thigh and quadriceps strength and volume are positively associated with testosterone levels, suggesting that training these muscle groups can improve sexual function.^27^ Additionally, pelvic floor muscles play a crucial role in maintaining erectile hardness.^28^ Effective contractions of these muscles can prevent venous outflow from the penis, thereby enhancing and maintaining penile rigidity.^29^ Consequently, many male fitness enthusiasts enhance sexual function through leg and pelvic floor muscle exercises. Our findings also indicate that a slower walking pace increases the risk of ED, whereas hand grip strength shows no causal relationship. However, it is crucial to interpret walking pace not merely as a proxy for physical muscle strength, but as a highly integrative functional trait. Walking pace reflects an individual’s overall cardiorespiratory fitness, neurological integrity, and cardiovascular health. These physiological systems share overlapping pathophysiological pathways with ED. The causal association observed between slower walking pace and ED should not be attributed solely to direct muscular causality, but rather recognized as a reflection of physical, neurological, and vascular decline.

This study is the first to employ a bi-directional 2-sample MR analysis to investigate the causal relationship between sarcopenia-related traits and ED. Our findings were validated through sensitivity analyses and pleiotropy assessments. The study has several strengths, such as effectively reducing bias from confounding factors, thereby increasing the credibility of the results. Investigating the underlying mechanisms of ED is crucial as it may provide new insights for early diagnosis and treatment. However, our study also has some limitations. First, the MR analysis is based on European samples, which means our results may not be generalizable to other populations. Second, due to the limitations of the original data, we were unable to perform stratified analyses based on different exposure levels, age groups, or ED severity. Therefore, more detailed studies are needed in the future to confirm our conclusions. Additionally, ED encompasses various subtypes, such as vascular and neurogenic ED. Due to the lack of specific subtype data, we could not assess the relationship between sarcopenia-related traits and specific ED subtypes. Lastly, the three traits we investigated might not fully represent sarcopenia. Thus, it is crucial to expand the sample size and include diverse populations in future studies. Further in vivo and in vitro experiments are also needed to verify our conclusions.

## Conclusion

This MR study suggests that specific sarcopenia-related functional traits, particularly slower walking pace, may play a causal role in erectile dysfunction, whereas reverse causality was not observed. These findings highlight the importance of physical function and overall fitness in ED risk, although further studies are needed to clarify the role of muscle mass and to validate these results across diverse populations.

## Supplementary Material

supplementary_figure_qfag010

CONSORT_2010_Checklist_refuerzo_qfag010

JSM-25-1140_R1-Backmatter_qfag010

## Data Availability

All data generated or during this study are included in this published article. All GWAS data for this study can be downloaded from the online database.
