# Supplementary figures and images for: Sarcopenia-related traits and erectile dysfunction: a bi-directional Mendelian randomization study
Source: Sex Med. 2026 Mar 27;14(2):qfag010. doi: 10.1093/sexmed/qfag010 (PMC13025071; doi:10.1093/sexmed/qfag010)

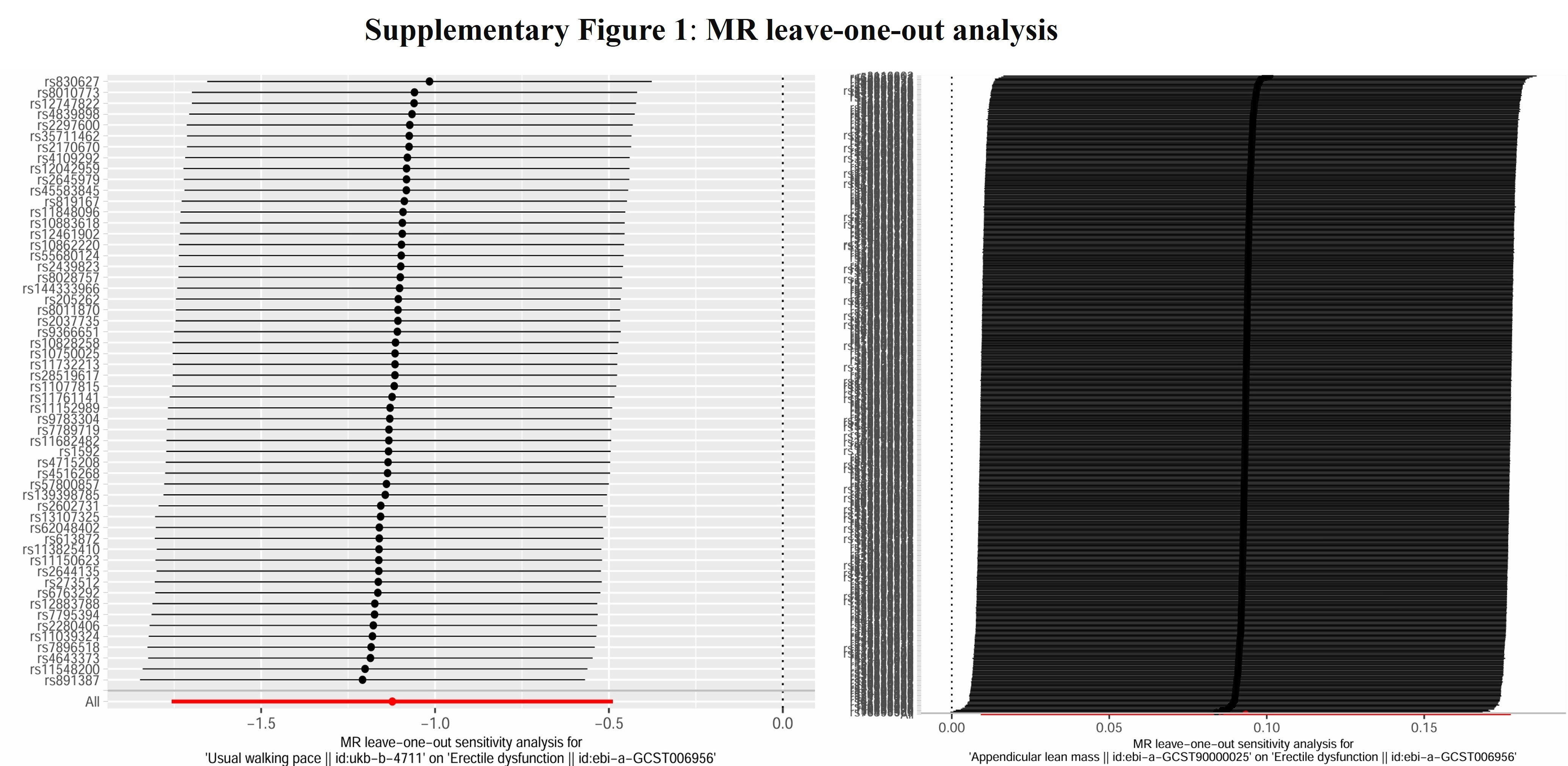

Supplement: supplementary_figure_qfag010 [file supplementary_figure_qfag010.jpeg]
