# Supplementary material for: Sarcopenia-related traits and erectile dysfunction: a bi-directional Mendelian randomization study
Source: Sex Med. 2026 Mar 27;14(2):qfag010. doi: 10.1093/sexmed/qfag010 (PMC13025071; doi:10.1093/sexmed/qfag010)
Supplement: JSM-25-1140_R1-Backmatter_qfag010 [file jsm-25-1140_r1-backmatter_qfag010.docx]

**Acknowledgements**

We thank all doctors, therapists, nursing staff, and administrative personnel who supported patient recruitment, treatment, and follow-up in Colombia and Mexico.

**Author contribution**

Conceptualization: C.S-S, H.C, and JM. Data curation: C.S-S. Formal analysis: C.S-S. Investigation: H.C, J.M, J.B and F.P.  Methodology: C.S-S. Writing – original draft: C.S-S, H.C, and J.M. Writing – review & editing: J.B and F.P. Final approval of the completed article: C.S-S, J.M, H.C, J.B and F.P

**Clinical Trial Registration:** ClinicalTrials.gov, NCT03308409.

**Ethical approval**

The study was approved by the Ethics Committee of the Bioethics Institute of the Pontificia Universidad Javeriana in Colombia (Remission 21-07-2017) and by the Ethics and Research Committee for human studies of Médica Sur in Mexico (Conbioética-09-CEI-018-20160729).
